# COMPARE CPM-RMI Trial: Intramyocardial Transplantation of Autologous Bone Marrow-Derived CD133^+^ Cells and MNCs during CABG in Patients with Recent MI: A Phase II/III, Multicenter, Placebo-Controlled, Randomized, Double-Blind Clinical Trial 

**DOI:** 10.22074/cellj.2018.5197

**Published:** 2018-03-18

**Authors:** Mohammad Hassan Naseri, Hoda Madani, Seyed Hossein Ahmadi Tafti, Maryam Moshkani Farahani, Davood Kazemi Saleh, Hossein Hosseinnejad, Saeid Hosseini, Sepideh Hekmat, Zargham Hossein Ahmadi, Majid Dehghani, Alireza Saadat, Soura Mardpour, Seyedeh Esmat Hosseini, Maryam Esmaeilzadeh, Hakimeh Sadeghian, Gholamreza Bahoush, Ali Bassi, Ahmad Amin, Roghayeh Fazeli, Yaser Sharafi, Leila Arab, Mansour Movahhed, Saeid Davaran, Narges Ramezanzadeh, Azam Kouhkan, Ali Hezavehei, Mehrnaz Namiri, Fahimeh Kashfi, Ali Akhlaghi, Fattah Sotoodehnejadnematalahi, Ahmad Vosough Dizaji, Hamid Gourabi, Naeema Syedi, Abdolhosein Shahverdi, Hossein Baharvand, Nasser Aghdami

**Affiliations:** 1 Department of Surgery, Baqiyatallah Hospital, Tehran, Iran; 2 Department of Regenerative Medicine, Cell Science Research Center, Royan Institute for Stem Cell Biology and Technology, ACECR, Tehran, Iran; 3 Research Department, Tehran Heart Center, Tehran University of Medical Sciences, Tehran, Iran; 4 Department of Echocardiography, Baqiyatallah Hospital, Tehran, Iran; 5 Department of Cardiology, Baqiyatallah Hospital, Tehran, Iran; 6 Department of Cardiac Surgery, Lavasani Hospital, Social Security Organization, Tehran, Iran; 7 Rajaie Cardiovascular Medical and Research Center, Iran University of Medical Sciences, Tehran, Iran; 8 Department of Nuclear Medicine, Hasheminejad Hospital, Tehran University of Medical Sciences, Tehran, Iran; 9 Transplantation Research Center, NRITLD, Masih Daneshvari Hospital, Shaheed Beheshti University of Medical Science, Darabad, Niavaran, Tehran, Iran; 10 Department of Internal Medicine, Baqiyatallah Hospital, T ehran, Iran; 11 Student Research Committee, School of Nursing and Midwifery , Shahid Beheshti University of Medical Sciences, Tehran, Iran; 12 Echocardiography Research Center, Rajaie Cardiovascular Medical and Research Center , Iran University of Medical Sciences, Tehran, Iran; 13 Department of Pediatrics, Ali Asghar Pediatric Hospital, Tehran University of Medical Sciences, Tehran, Iran; 14 Department of Hematology and Oncology, Firoozgar Hospital, Iran University of Medical Sciences, Tehran, Iran; 15 Department of Heart Failure and Transplantation, Fellowship in Heart Failure and Transplantation, Rajaie Cardiovascular Medical and Research Center, Iran University of Medical Sciences, Tehran, Iran; 16 Department of Internal Medicine, Lavasani Hospital, Social Security Organization, Tehran, Iran; 17 Department of Epidemiology and Reproductive Health, Reproductive Epidemiology Research Center, Royan Institute for Reproductive Biomedicine, ACECR, Tehran, Iran; 18 Department of Reproductive Imaging, Reproductive Biomedicine Research Center, Royan Institute for Reproductive Biomedicine, ACECR, Tehran, Iran; 19 Department of Genetics, Reproductive Biomedicine Research Center, Royan Institute for Reproductive Biomedicine, ACECR, Tehran, Iran; 20 School of Pharmacy and Medical Sciences, Sansom Institute for Health Research, University of South Australia, South Australia, Australia

**Keywords:** Autologous Transplantation, Bone Marrow-Cells, Cell Therapy, Mononuclear Cells, Myocardial Infarction

## Abstract

**Objective:**

The regenerative potential of bone marrow-derived mononuclear cells (MNCs) and CD133^+^ stem cells in the heart varies in terms of their pro-angiogenic effects. This phase II/III, multicenter and double-blind trial is designed to compare the functional effects of intramyocardial autologous transplantation of both cell types and placebo in patients with recent myocardial infarction (RMI) post-coronary artery bypass graft.

**Materials and Methods:**

This was a phase II/III, randomized, double-blind, placebo-controlled trial COMPARE CPM-RMI (CD133, Placebo, MNCs - recent myocardial infarction) conducted in accordance with the Declaration of Helsinki that assessed the safety and efficacy of CD133 and MNCs compared to placebo in patients with RMI. We randomly assigned 77 eligible RMI patients selected from 5 hospitals to receive CD133^+^ cells, MNC, or a placebo. Patients underwent gated single photon emission computed tomography assessments at 6 and 18 months post-intramyocardial transplantation. We tested the normally distributed efficacy outcomes with a mixed analysis of variance model that used the entire data set of baseline and between-group comparisons as well as within subject (time) and group×time interaction terms.

**Results:**

There were no related serious adverse events reported. The intramyocardial transplantation of both cell types increased left ventricular ejection fraction by 9% [95% confidence intervals (CI): 2.14% to 15.78%, P=0.01] and improved decreased systolic wall thickening by -3.7 (95% CI: -7.07 to -0.42, P=0.03). The CD133 group showed significantly decreased non-viable segments by 75% (P=0.001) compared to the placebo and 60% (P=0.01) compared to the MNC group. We observed this improvement at both the 6- and 18-month time points.

**Conclusion:**

Intramyocardial injections of CD133^+^ cells or MNCs appeared to be safe and efficient with superiority of CD133+ cells for patients with RMI. Although the sample size precluded a definitive statement about clinical outcomes, these results have provided the basis for larger studies to confirm definitive evidence about the efficacy of these cell types (Registration Number: NCT01167751).

## Introduction

Autologous bone marrow-derived cell therapy is under current investigation as a potentially promising therapy to treat patients with ischemic heart disease and potential candidates for revascularization with coronary artery bypass grafts (CABG) (1). The goal of this treatment is to improve myocardial regeneration and angiogenesis through administration of therapeutic cells into the periinfarct areas of the ischemic myocardium. Mononuclear cells (MNCs) (2-6) and CD133^+^ cells (7-18) are two major bone marrow-derived cells used as potential treatments for ischemic heart diseases. However, some studies report favorable outcomes whereas others indicate no benefits. These discrepancies may be related to factors such as the numbers of injected cells, administration route, time interval from myocardial infarction (MI), type of injected cells, cell isolation and preparation methods, and assessment techniques that include echocardiography, single photon emission computed tomography (SPECT), and magnetic resonance imaging (MRI). Nevertheless, these types of cells are easy to harvest, simple to administer, ethically acceptable, and do not require immunosuppression (19). 

CD133^+^ bone marrow hematopoietic stem cells possess the characteristics of endothelial progenitor cells. These cells have the capability to differentiate into endothelial cells in vitro and play a role in neoangiogenesis processes in vivo (20, 21). Compared to non-selected bone marrow mononuclear cells, CD133^+^ cells have greater proangiogenic effects due to secretion of related cytokines, graft-host cell interactions (22-24), and resistance to apoptosis (25). The efficacy of intramyocardial injection of bone marrow-derived CD133^+^ cells versus MNCs in restoring function to an injured myocardium within an established infarct, however, has not been explored.

We sought to determine the functional consequences and clinical events that followed direct intramyocardial delivery of autologous bone marrow-derived MNCs and CD133^+^ cells in MI patients in this phase II/III multicenter, randomized, double-blind, placebo-controlled study. Findings from a comparison of CD133^+^ cells or MNCs versus placebo in the COMPARE CPM-RMI (CD133, Placebo, MNCs)-(recent myocardial infarction) trial have implications for the development of cell-based therapies for ischemic heart failure.

## Materials and Methods

### Study design, enrollment and patient population

We conducted the COMPARE CPM-RMI phase II/III, randomized, double-blind, placebo-controlled trial of the safety and efficacy of the cell procedure in accordance with the Declaration of Helsinki. This study was performed in 5 Tehran, Iran hospitals (Baqiyatallah, Shahid Dr. Lavasani, Tehran Heart Center, Rajaie Cardiovascular Medical and Research Center, and Masih Daneshvari). The patients’ documentations were collected from Royan Institute and the appropriate, related hospital. This study received approval from the Ethical Committee of Royan Institute (reference number: p-85-106). This trial was registered at http://www.Clinicaltrials.gov (identifier: NCT01167751). All patients gave written informed consent. 

**Fig.1:**
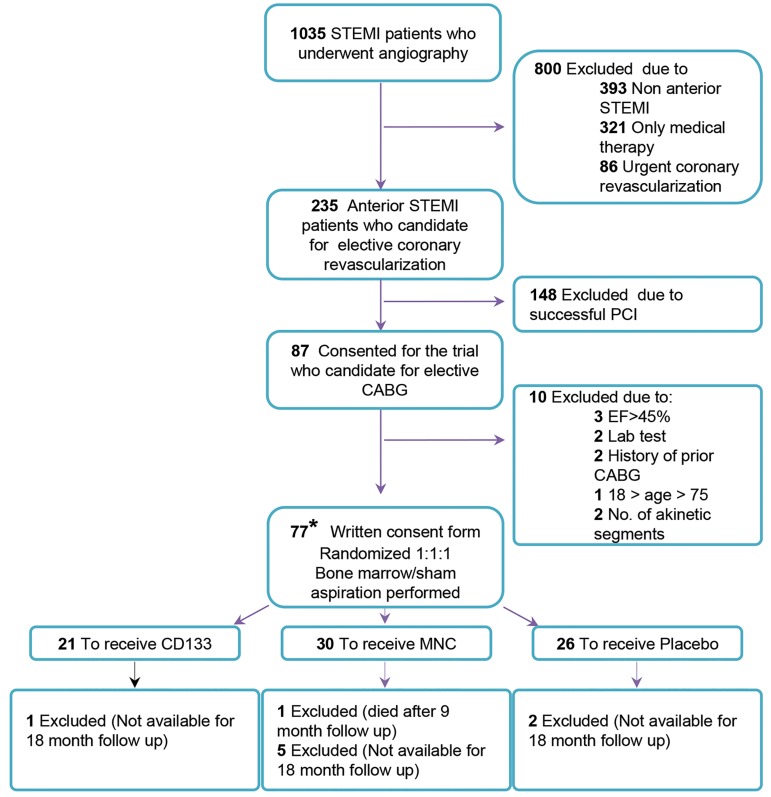
Flow chart for patient eligibility. We selected patients with STEMI at defined intervals from 10 days to 3 months. Ineligible patients were excluded by angiography results among other criteria as mentioned in the diagram. We divided eligible patients into three groups: placebo, MNC, and CD133^+^. All patients were intramyocardly injected 2 cc of suspension in 10 points of the marginal zone of the infarcted muscle during open heart surgery and were followed by SPECT scan at 6 and 18 months. From these patients, 8 were not available for follow up and one patient from the MNC group died. STEMI; ST elevation myocardial infarction, CABG; Coronary artery bypass graft, PCI; Percutaneous coronary intervention, LVEF; Left ventricular ejection fraction, MNC; Mononuclear cells, SPECT; Single photon emission computed tomography, and *; According to sample size calculation, we considered 90 patients for enrollment, however financial limitations forced us to stop patient recruitment. This study enrolled and randomly assigned 77 patients to three groups: MNC, CD133^+^, and placebo.

Patients were randomized at Royan Institute beginning in January 2008 with follow-up visits completed in July 2012. The flow chart shows patient eligibility (Fig .1). We selected 1035 patients recently diagnosed with first ST-elevation myocardial infarction (STEMI). The inclusion and exclusion criteria is listed in detail (Table 1). Patients aged 18 to 75 years received standard therapy and were chosen according to a major two-step selection process. Initially, each patient underwent an angiography evaluation that determined their eligibility for elective CABG. For the second step of the selection process, each patient underwent an eligible criteria assessment comprised of laboratory tests, dobutamine stress echocardiography, and SPECT analysis for baseline characteristics. Finally, we randomized 77 eligible patients via a computer-generated randomization sequence in a 1:1:1 ratio between the CD133^+^, the MNC, and placebo groups. Patients underwent bone marrow or sham aspirations. Patients and investigators not affiliated with the cell-processing laboratory were blinded to preparation and administration of the study product. Each patient had follow up visits at 6 and 18 months after transplantation. 

**Table 1:** Patient inclusion and exclusion criteria


Inclusion criteria	
	Age: 18-75 Y
	First ST elevation myocardial infarction (STEMI) 10 days -3 months
	Post-acute myocardial infarction (AMI) left ventricular ejection fraction (LVEF) as assessed by echocardiography: 20-45%
	Target lesion must be located in the left anterior descending (LAD) section
	Coronary artery bypass graft (CABG) candidate
	At least 4 akinetic segments (assessed by stress echocardiography)
Exclusion criteria	
	History of prior AMI
	Active infection, history of recurrent infection, positive test for syphilis (RPR), hepatitis B and C (HBsAg, anti- HBc, anti-HCV), HIV or HTLV-1
	Documented terminal illness or malignancy
	Documented autoimmune disease
	Pulmonary edema
	Urgent or emergency CABG
	Systolic blood pressure<80 mmHg
	4000>leukocytes (µL)>15000
	AST>100 IU/L or ALT>100 IU/L
	Hemoglobin<10 g/dL
	Platelets<100,000/µL
	INR>1.8


HBsAg; Hepatitis B surface antigen, HBc; Hepatitis B core, HCV; Hepatitis C virus, HIV; Human immunodeficiency virus, HTLV-1; Human T-cell lymphotropic virus type 1, AST; Aspartate aminotransferase, ALT; Alanine aminotransferase, and INR; International normalized ratio.

### Study procedures and timeline

Baseline information included results of chemistry, hematology and virology laboratory tests, in addition to SPECT and stress echocardiography. We conducted patient interviews to determine demographic and clinical variables. 

**Fig.2:**
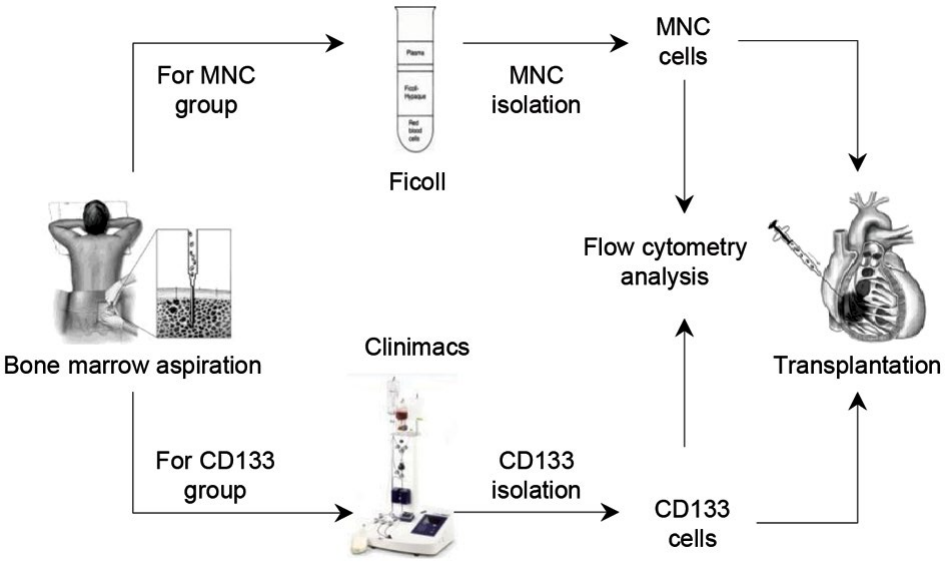
MNCs and CD133^+^ cells isolation and transplantation protocols for CABG candidates. Patients underwent bone marrow aspiration 24 hours before CABG. MNCs and CD133^+^ cells were isolated by Ficoll density gradient and a CliniMACS machine. Isolated cells were analyzed by flow cytometry before intramyocardial injection. Patients in the placebo group underwent a sham aspiration procedure. CABG; Coronary artery bypass grafts and MNC; Mononuclear cells.

One day prior to surgery, patients in the CD133^+^ and MNC groups underwent bone marrow aspiration (150 ml) from their iliac crests. The isolation of CD133^+^ cells and MNCs will be described in a later section. The cells were suspended in 2 ml normal saline supplemented with 2% autologous serum. Placebo group patients underwent a sham aspiration procedure to ensure proper blinding of the treatment assignment. The sham procedure consisted of creating a skin incision under local anesthesia at the iliac crest surface. In the placebo group, patients received vehicle (2 ml) comprised of normal saline supplemented with 2% autologous serum. The MNC and CD133^+^ cell isolation and transplantation protocol is summarized for the two cell therapy groups (Fig .2).

Patients from all study groups received the injections at the end of the cardiopulmonary bypass and cold blood cardioplegic arrest, and just prior to removal of the aortic cross clamp. Patients received either cells or vehicle placebo as intramyocardial injections to 10 sites (0.2 ml per site) at the marginal zone of the subepicardial infarcted area using a 22-gauge needle. We performed primary echocardiography and SPECT, as the paraclinical imagings, to locate the infarcted area. During the procedure, surgeons visually estimated the border zone of the infarct area and performed the injections. Lists The mean numbers of injected CD133^+^ and MNC cells were shown for these groups (Table 2). The average number of injected cells in the 2 ml cell suspension was 564.63×10^6^ (± 69.35) MNC cells and 8.19×10^6^ (± 4.26) CD133^+^ cells per patient. Each patient received 1/10 of each cell suspension injected per site. After the myocardial cell injections, the area was gently massaged, followed by reperfusion. After the CABG, all patients remained hospitalized for a minimum of 4 days. Patients were evaluated at 1 week after CABG, then monthly for 3 months, and at 6 and 18 months. At the long-term (6 and 18 months) follow- ups, we assessed for safety and efficacy that included a clinical interview of the New York Heart Association (NYHA) Classification, physical examination, and cardiac SPECT.

**Table 2:** Average number of cells and immunophenotyping of the CD133+ and MNC groups


Characteristics/patient	MNC groupn=30Mean (SD)	CD133^+^ groupn=21Mean (SD)

Bone marrow cell number	2.11×10^9^(1.62)	2.39×10^9^(1.42)
Total injected cell number	564.63×10^6^ (69.35)	8.19×10^6^ (4.26)
Injected cell number/site	564.63×10^5^ (69.35)	8.19×10^5^ (4.26)
Viability (%)	98.53 (1.67)	92.91 (7.66)
CD133^+^ (%)	1.19 (1.21)	63.69 (17.84)
CD34^+^(%)	0.20 (0.24)	n.d.
CD44^+^(%)	88.16 (17.57)	n.d.
CD31^+^ (%)	42.77 (18.98)	n.d.
VEGFR (%)	38.60 (26.18)	n.d.


CD; Cluster of differentiation, MNC; Mononuclear cells, n.d.: Not determined, and VEGFR; Vascular endothelial growth factor receptor.

### Objectives and endpoints

This study focused on improvement of myocardial perfusion and left ventricular (LV) function in STEMI patients after injections of bone marrow derived cells into the infarcted myocardium compared to a placebo group. Additionally, we evaluated different effects of CD133^+^ and MNC cells at determined endpoints. The primary endpoint was assessed by changes in global LV ejection fraction (LVEF) at rest by gated SPECT. 

Secondary endpoints were: i. Adverse cardiac events that included death, reinfarction, implantable cardioverter defibrillator (ICD) placement, infection, and arrhythmia, ii. Changes in wall motion score (WMS), decreased systolic wall thickening (Dec. Thickening) of the myocardium, non-viable (NV) segments, and perfusion defect score (PDS) assessed by gated SPECT, and iii. NYHA classification. We compared and analyzed endpoint data amongst the three groups during the 18 months of follow up. 

### Cell separation and flow cytometry

We isolated (150 ml) bone marrow MNCs under Good Manufacturing Practice (GMP) conditions. Bone marrow MNCs were counted by a NucleoCounter instrument (ChemoMetec A/S, Denmark). We isolated the MNCs by centrifuging the resultant bone marrow through a low-density gradient using Ficoll-Paque PREMIUM (Lymphodex, Inno-train, 002041600) according to the manufacturer’s protocol. Briefly, bone marrow samples [diluted 1:1 in phosphate- buffered saline (PBS)] were subsequently loaded over a Ficoll-Paque at a 3:1 ratio and centrifuged for 30 minutes at 400 g with no brake. Cells were collected at the interface and washed in PBS buffer (1:1; Milteny Biotech GmbH, 700-25). Cell viability and counts were determined by trypan blue exclusion staining and a NucleoCounter. 

CD133^+^ cells were isolated by a CliniMACS automated machine. The bone marrow cell suspension was filtered through a 200 µm filter (Baxter S.A, RMC 5849). We used a 10% Gamunex reagent as the blocking antibody (100 mg/ml, Talecris Biotherapeutics, Inc.). A monoclonal magnetic micro-bead antibody that had the capability to detect CD133^+^ cells (Milteny Biotech GmbH, 172-01) was applied. The magnetically labeled cell bags were subsequently attached to a CliniMACS tubing set LS (Milltenyi Biotec GmbH) and sorted by a CliniMACS instrument. We assessed cell count, viability, and sterility, after which the positive fractions were centrifuged for 5 minutes at 350 g.

We assessed for cell specific marker expressions in the cell populations by flow cytometry. Cells were stained with phycoerythrin (PE) or fluorescein isothiocyanate (FITC) conjugated anti- human CD133 (293C3, Milteny Biotech GmbH, 130090- 853), hVEGFR2/KDR (R&D Systems, FAB357P), CD31 (BD Biosciences, 555445), CD45/34 (BD Biosciences, 341071), and CD44 (BD Biosciences, 555479) antibodies, IgG2b/RPE (BD Biosciences, 556656), and IgG1FITC/IgG1RPE (Dako, X0932). Next, they were analyzed with a BD FACSCalibur flow cytometry system (BD Biosciences, San Jose, CA, USA) and software version 2.5.1 (BD Biosciences) by gating at 2% of the isotype control. A total of 100,000 events were acquired for each marker and isotype.

### Single photon emission computed tomography and stress echocardiography imaging

We performed SPECT analysis at baseline, then at 6 and 18 months after transplantation. SPECT images were obtained approximately one hour after an intravenous injection of 740-925 MBq of 99mTc-sestamibi by a single-head camera with the following parameters: low- energy all-purpose (LEAP) collimator, 20% symmetric window at 140 keV, and a 64×64 matrix. Step and shoot acquisition and projection images were reconstructed. We used AutoQUANT software version 4.3.1 (AutoQUANT, ADAC Laboratories, Milpitas, CA) to evaluate PDS, LVEF, WMS, NV, and Dec. Thickening according to a scoring method outlined in Figure S1 (See Supplementary Online Information at www.celljournal.org) (26) that follows a model of 17 cardiac segments. We have assessed the PDS variable according to 4 scores: mild hypoperfusion (1), moderate hypoperfusion (2), severe hypoperfusion (3), and absence of perfusion (4). The NV score (2) segments were important for viability assessment. The 3 scores for abnormal movement included hypokinesia (1), akinesia (2), and dyskinesia (3). We assessed Dec. Thickening as mild (1), moderate (2), or severe (3). 

Patients received intravenous injections of low-dose dobutamine for stress echocardiography. The stress echocardiography images were obtained from the parasternal (long and short axis) in the left lateral decubitus and apical (2 and 4 chamber) windows. Five variables that included the LVEF, left ventricular end diastolic diameter (LVEDD), LVE systolic diameter (LVESD), the number of NV segments, and WMS index were evaluated using a 17-segment model from standard parasternal and apical 2D echocardiography recommended by the American Society for Echocardiography. A total of 5 expert echocardiographers blinded to the patient group assignments performed the measurements with the same machine type (GE Vivid 7 with a 3 MHz probe) in each of the 5 centers.

### Statistical analysis

We extracted the confidence intervals (CIs) of outcomes to evaluate improvements in myocardial perfusion and LV function in study patients treated with CD133^+^ cells or MNC versus placebo and in patients that received CD133^+^ cells versus MNC. All statistical analyses were performed by taking into consideration the intention to treat (ITT) point of view. We used analysis of variance (ANOVA) for quantitative outcomes and the χ2 test for qualitative baseline variables to analyze for differences between the study groups. Normal distributed efficacy outcomes were tested with a mixed analysis of variance model that used the entire data set of baseline, between- group comparisons, within subject (time), and group×time interactions (27, 28). 

The NYHA class change scores (baseline vs. 18 months) were calculated to ascertain treatment efficacy by the χ2 test. The sample size was estimated from a study by Zhao et al. (29) and conservative assumptions. At least 27 patients were considered adequate for each group; according to the potential for patient loss during the trial, we determined the total sample size as 90 with a power of >80%. Data analyses were performed by the repeated measures method, standard deviation to 9 units, and a minimum clinically important difference to 6 units in the LVEF scale as a primary end-point, with a correlation between 6 and 18 months to 0.6. We assumed 6 unit differences only for the cell therapy groups against the placebo. Analyses were conducted using SPSS software version 16 (SPSS, Chicago, IL, USA). The data have been presented as mean ± SD. A P<0.05 was considered statistically significant.

## Results

### Patients

We considered an enrollment of 90 patients according to the sample size calculation. However financial limitations forced us to stop patient recruitment. Therefore, this study enrolled and randomly assigned 77 patients to three groups: MNC, CD133^+^ and placebo through anterior STEMI at a defined time interval. After opening the study code, we determined that the numbers of patients per group were not similar: MNC (n=30), CD133^+^ (n=21) and placebo (n=26). There were no significant differences in baseline characteristics detected between groups except for coronary artery disease, LVESD, and LVEDD. The study population was predominantly male (Table 3). 

There were 9 patients who received the study intervention but did not complete the 18 months of follow-up. One patient in the CD133^+^ and one in the MNC group were lost to follow up due to relocation out of the province. Four patients in MNC group and two in the placebo group were lost to follow up due to lack of telephone access. One patient from the MNC group died in the ninth month after surgery (Fig .1).

The average number of bone marrow MNC cells obtained was 2.11×10^9^ (± 1.62) in the MNC group and 2.39×10^9^ (± 1.42) in the CD133^+^ group (Table 2). The average number of injected cells prepared in a 2 ml cell suspension of normal saline with 2% autologous serum was 564.63×10^6^ (± 69.35) MNC cells and 8.19×10^6^ (± 4.26) CD133^+^ cells per recipient.

### Safety and adverse events during follow-up

There were no reported study related serious adverse events during the initial hospitalization. However, 2 patients in the placebo group experienced reinfarction at 8 and 11 months after surgery. Additionally, 2 patients who had an LVEF <30% and mild-to-moderate symptoms of heart failure in the MNC group agreed to undergo ICD implantation as a primary prevention at the 6- and 18-month post-surgery follow up visits as recommended by the ACC/AHA Guidelines (30). One patient in MNC group died from cardiac arrest during the 9th month after surgery. There was no autopsy performed for this patient. Additionally, we observed no infections or arrhythmias among patients from all groups. The number of total adverse events per group were: placebo [2], CD133^+^ [0], and MNC [3].

### Physical functional 

The NYHA class improved after 18 months compared with baseline in 17 (85.0%) CD133^+^ patients, 18 (75.0%) MNC patients, and 13 (54.2%) placebo patients. There was no change in 3 (15.0%) CD133^+^ patients, 6 (25.0%) MNC patients, and 10 (41.7%) placebo patients. There was no worsening in NYHA classification among the CD133^+^ and MNC patients after 18 months. However, one patient from the placebo group had an increase in the NYHA classification (P=0.178, Table S1) (See Supplementary Online Information at www.celljournal.org).

**Table 3:** Patient baseline characteristics


Parameter	Placebo group n=26	CD133+ group n=21	MNC group n=30	P value

Age (Y), mean (SD)	55.50 (8.54)	53.14 (8.56)	51.45 (7.49)	NS
Females, n (%)	3 (11.5)	2 (9.5)	3 (10)	NS
Body mass index, mean (SD)	26.7 (4.2)	27.9 (2.6)	26.0 (2.7)	NS
Duration*, (median days)	32.5	30	26.5	NS
Min-Max (day)	10-79	10-90	10-76	
CAD, n (%)				0.039
LAD only	2 (7.6)	0.0	5 (16.6)	
LAD+RCA/LCX	4 (15.3)	6 (28.5)	11 (36.6)	
LAD+RCA+LCX	20 (76.9)	15 (71.4)	14 (46.6)	
Diabetes mellitus, n (%)	10 (38.4)	6 (28.5)	11 (36.6)	NS
Hypertension, n (%)	6 (23.0)	7 (33.3)	6 (20.0)	NS
Hyperlipidemia, n (%)	14 (53.8)	9 (42.8)	15 (50.0)	NS
Smoking, n (%)	12 (46.1)	7 (33.3)	15 (50.0)	NS
Preoperative medication, n				NS
Aspirin	22	16	24	
Clopidogrel	13	11	16	
Βeta-blockers	24	16	23	
ACE inhibitors	13	12	20	
Statins	22	18	21	
Nitrates	20	14	8	
Diuretic	10	6	8	
Digoxin	9	8	7	
Thrombolytic	12	10	14	
Lab test (U/L) [mean (SD)]				NS
Peak CK-total	774 (541)	924 (301)	646 (310)	
Peak CK-MB	52 (37)	54 (24)	76 (74)	
SPECT [mean (SD)]				NS
LVEF (%)	40.47 (15.12)	39.80 (10.63)	37.06 (9.01)	
PDS	10.82 (9.24)	17.10 (10.25)	16.38 (9.98)	
NV	2.05 (3.12)	4.14 (3.65)	3.00 (3.31)	
WMS	5.11 (3.62)	5.50 (3.74)	6.93 (5.00)	
Dec. Thickening	8.64 (6.86)	11.10 (4.25)	14.46 (8.55)	
S. echo, mean (SD)				
LVEF (%)	32.90 (8.40)	31.13 (5.37)	32.34 (5.86)	NS
NV	5.83 (0.77)	5.96 (0.75)	6.32 (0.68)	NS
WMSI	2.00 (0.09)	2.14 (0.11)	1.96 (0.07)	NS
LVEDD (mm)	55.60 (6.37)	59.19 (7.03)	53.08 (5.36)	0.012
LVESD (mm)	43.98 (8.50)	46.65 (7.78)	40.68 (6.36)	0.050
Operative details				NS
Number of grafts, mean (SD)	3.6 (0.7)	3.5 (0.8)	3.2 (0.8)	
Lima grafts, n	26	20	30	
X-clamp time (minute)	39 (10)	46 (16)	45 (13)	
Perfusion time (minute)	68 (18)	74 (27)	73 (21)	


MNC; Mononuclear cells, CAD; Coronary artery disease, LAD; Left anterior descending, RCA; Right coronary artery, LCX; Left circumflex artery, SPECT; Single photon emission computed tomography, LVEF; Left ventricular ejection fraction, PDS; Perfusion defect score, NV; Non-viable segments, WMS; Wall motion score, Dec. Thickening; Decreased systolic wall thickening, S. echo; Stress echocardiography, WMSI; Wall motion score index, LVEDD; Left ventricular end diastolic diameter, LVESD; Left ventricular end systolic diameter, NS; Not significant, *; Days from myocardial infarction (MI) to coronary artery bypass graft (CABG).

### Global function

**Fig.3:**
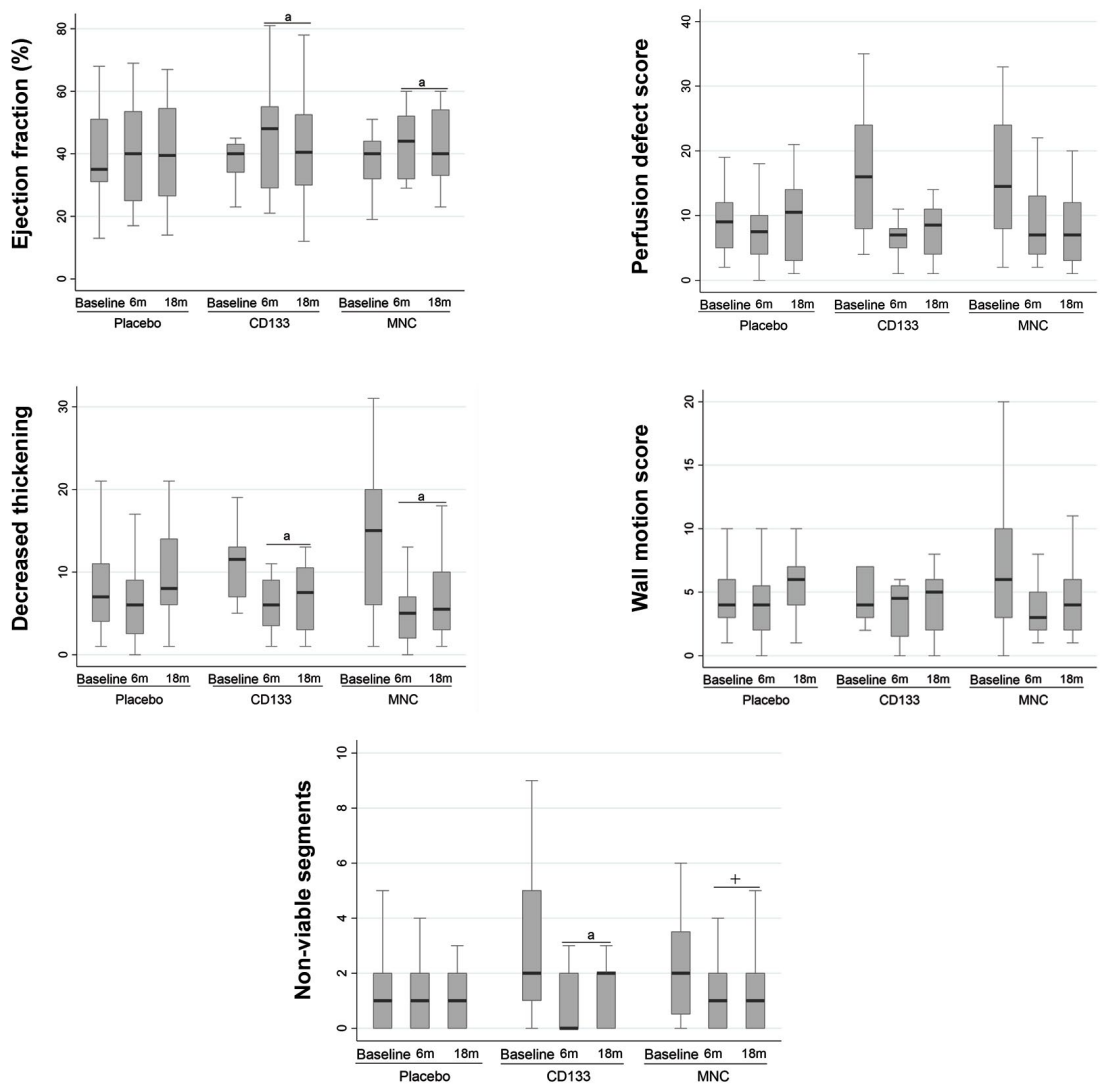
Comparing end-point analyses of different variables between 6 and 18 months by SPECT. Significant differences existed between the cell therapy and placebogroups in ejection fraction, non-viable segments and decreased systolic wall thickening variables as measured by SPECT. There was no time effect observed. Therefore, we considered statistical significance for both the 6- and 18-month time periods. Significant difference between the cell and placebo groups. a; P<0.03, CD133^+^ or MNC groups versus placebo, +; P<0.01, significant difference between CD133^+^ and MNC groups. Data markers represent means + SD, and 95% CIs. ANOVA was conducted with repeated measures. CI; Confidence interval, MNC; Mononuclear cells, and SPECT; Single photon emission computed tomography.

We used cardiac SPECT to assess LVEF, NV segments, Dec. Thickening, WMS, and PDS at baseline, and 6 and 18 months after transplantation (Fig .3, Table S2) (See Supplementary Online Information at www.celljournal. org). Analysis of the parameters showed consistent results at both time points. LVEF increased by 9% in CD133^+^ group (95% CI: 2.14% to 15.78%, P=0.011) and 7% in the MNC group (95% CI: 1.05% to 12.79%, P=0.022) relative to the placebo group. 

NV segments decreased by 75% equals to -1.5 (95% CI:-2.33 to-0.62, P=0.001) after administration of CD133^+^ cells, yet remained unchanged with MNC -0.30 (95% CI:-1.09 to 0.48, P=0.443) compared to the placebo. Additionally, the NV segments in the CD133^+^ group significantly decreased 60% equals to -1.2 (95% CI:-2.06 to-0.29, P=0.010) compared to MNC group. 

During the 18 months follow up, we observed significantly improved Dec. Thickening in both the CD133^+^ and MNC groups with a mean change of -3.75 (95% CI:-7.07 to-0.42, P=0.028) for the CD133^+^ group and -3.55 (95% CI:-6.61 to-0.49, P=0.024) for the MNC group. These improvements remained consistent up to the 18-month follow up (P<0.05). Significant differences existed between the MNC and placebo groups in LVEF and Dec. thickening. We observed comparable results in the CD133^+^ versus placebo groups. Additionally, there were no significant differences between the CD133^+^ and MNC groups except in NV segments in CD133^+^ group. 

We assessed the correlations of baseline factors (smoking, diabetes mellitus, hyperlipidemia, and hypertension) with treatment response in all three groups. Patients who smoked in all three groups showed a 7% decrease in LVEF (P=0.046). We observed no correlation in the other variables. 

## Discussion

Clinical trials of intramyocardial bone marrow-derived cell injections have not reported any related serious adverse events, which this offers promise for MI (218). However, the efficacies of both MNCs and CD133^+^ cell transplantations remain uncertain. We designed the COMPARE CPM-RMI study with the intent to provide a placebo-controlled, double-blinded, safety and efficacy assessments of two leading candidates for cardiac cell therapy include bone marrow-derived non-selected MNCs and selected CD133^+^ cells. 

The MIXED model of the COMPARE CPM-RMI data suggested that CD133^+^ cells had slightly greater efficacy compared to MNCs. However, these findings were limited by the small sample size which resulted in preliminary conclusions. The variance-covariance matrix repeated measure analysis was assumed. Due to the type I error rate of 0.05% and observed differences in the treatment groups, 17 samples per arm were considered adequate to achieve an 80% power. Hence, the sample size and power of study were considered reliable despite the study limitations.

CD133^+^ cells decreased NV segments within the myocardium, which suggested a possible efficacy. The function of CD133^+^ cells in decreasing of NV segments and improving LVEF and systolic wall thickening remains to be determined. Results from animal studies show that transplanted cells integrate into the new environment, after which they form new vasculature and myocardium (11, 19). We hypothesize that this effect may be due to cytokine release, graft-host interactions (22-24), anti-apoptotic factors (25) and stimulation of neovascularization (20, 21) with cardiomyocyte regeneration primarily and by promotion of endogenous stem cell proliferation.

We did not observe any related serious adverse events from the intramyocardial cell injections. Since the first report on bone marrow-derived cell therapy for the treatment of heart failure in 2001 by Orlic et al. (31) numerous studies have used intracoronary, transendocardial, and/or intramyocardial injections. These trials indicated that the intramyocardial injection approach was the most efficient method due to direct visualization and delivery to the target area.

In this approach, the cells localize more reliably in the heart (32, 33). However, with intracoronary injections many of the injected cells eventually localize in the lungs or liver. 

MNCs had a mean number of 565×10^6^ injected cells per recipient compared to 8×10^6^ CD133^+^ cells per recipient. Although the number of injected CD133^+^ cells was comparable in both groups, where 6.72×10^6^ (1.19%) of the injected MNCs were CD133^+^ cells, we observed greater effectiveness with the enriched CD133^+^ cells. The heterogeneous population of the MNCs could affect homing of the desired cells, which was likely due to competition of multiple progenitors for engraftment and might reduce cell homing with well-known effects (34, 35). Previous human studies have shown that intracoronary transplantation of low numbers of enriched bone marrow progenitor cells have a 7-fold higher homing capacity compared to larger numbers of bone marrow-derived non- selected MNCs (36, 37).

On the other hand, the numbers of cells used for intracoronary cell therapy varied widely, even without taking into consideration trialsthat assessed the importance of cell number on clinical or functional outcome. We selected the number of CD133^+^ cells based on the positive effects of these cells that have been discussed by previous reports (8, 10, 11, 18) and our phase 1 clinical trial (16, 38). Although the therapeutic effects of at least 50 × 106 bone marrow-MNCs has been noted in previous meta-analyses (3, 39), the debate over the efficacy of the numbers of cells continues. Some meta-analyses have found no association between the number of cells delivered and the outcome (5). Therefore, the effective number of cells has yet to be determined (40, 41).

Appropriate timing of cell delivery following an MI on the course of heart function has yet to be determined. Previous research on the appropriate timing of MNCs administered by percutaneous coronary intervention to patients after an acute MI (AMI) showed no detectable effects on recovery of regional LV function (42-44). Our analysis also indicated no significant time-related improvements in the outcomes. 

We observed between-group efficacy improvements after analyses of several outcome measures for the CD133^+^ cells and MNCs versus placebo. However, comparison between the two cell types showed no significant differences in most cases, with the exception of the N.V segment variable. This finding indicated that the study was not powered to show definitive efficacy comparisons between cell types. Another limitation, due to ethical considerations, was the sham aspiration through a superficial skin incision instead of a bone marrow harvest for placebo treated patients. We attempted to mimic a bone marrow aspiration procedure by placing patients in the prone position where they experienced the sensation of needle pressure during administration of a local anesthetic in the hip area, other related issues, and the intramyocardial injection of a vehicle. MRI is the gold standard for cardiac function and morphology. However, due to limited access, our patients did not undergo MRI imaging. The data quality of an echocardiography is objective but may vary between individuals and centers (45). We took advantage of SPECT modality where the results were reported by one nuclear medicine specialist and double-checked by a second nuclear medicine specialist. Therefore, in order to strengthen our findings, studies with more participants in conjunction with powerful assessments such as MRI imaging is necessary.

## Conclusion

This preliminary COMPARE CPM-RMI study showed the safety and possible efficacy of delivering cell-therapy by intramyocardial injections in patients with RMI who underwent CABG. These results provided the basis for larger studies to perform definitive assessments of the efficacy of this approach.

## Supplementary Figure



## Supplementary PDF


