# Supplementary figures and images for: COMPARE CPM-RMI Trial: Intramyocardial Transplantation of Autologous Bone Marrow-Derived CD133+ Cells and MNCs during CABG in Patients with Recent MI: A Phase II/III, Multicenter, Placebo-Controlled, Randomized, Double-Blind Clinical Trial
Source: Cell J. 2018 Mar 18;20(2):267–77. doi: 10.22074/cellj.2018.5197 (PMC5893299; doi:10.22074/cellj.2018.5197)

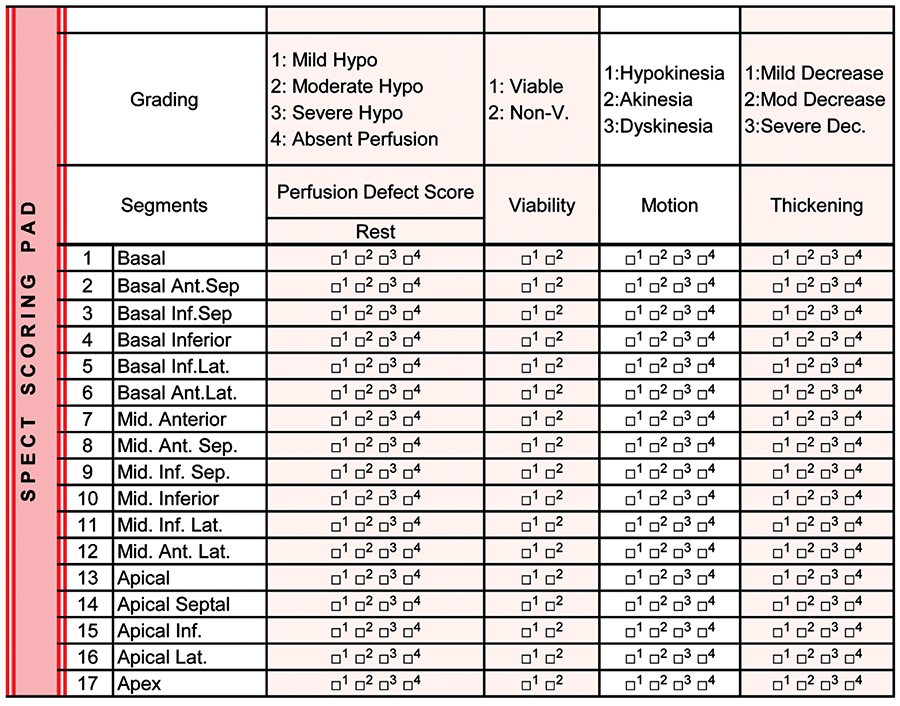

Supplement: Supplementary file 1 [file Cell-J-20-267-s01.tif]
