# Supplementary material for: COMPARE CPM-RMI Trial: Intramyocardial Transplantation of Autologous Bone Marrow-Derived CD133+ Cells and MNCs during CABG in Patients with Recent MI: A Phase II/III, Multicenter, Placebo-Controlled, Randomized, Double-Blind Clinical Trial
Source: Cell J. 2018 Mar 18;20(2):267–77. doi: 10.22074/cellj.2018.5197 (PMC5893299; doi:10.22074/cellj.2018.5197)
Supplement: Supplementary file 2 [file Cell-J-20-267-s02.pdf]

www.celljournal.org

Supplementary Information for

COMPARE CPM-RMI Trial: Intramyocardial Transplantation of Autologous Bone Marrow-Derived CD133+ Cells and MNCs during CABG in Patients with Recent MI: A Phase II/III, Multicenter, Placebo-Controlled, Randomized, Double-Blind Clinical Trial

Mohammad Hassan Naseri, M.D.[1#], Hoda Madani, M.D.[2#], Seyed Hossein Ahmadi Tafti, M.D.[3], Maryam Moshkani Farahani, M.D.[4], Davood Kazemi Saleh, M.D.[5], Hossein Hosseinnejad, M.D.[6], Saeid Hosseini, M.D.[7], Sepideh Hekmat, M.D.[8], Zargham Hossein Ahmadi, M.D.[9], Majid Dehghani, M.D.[6], Alireza Saadat, M.D.[10], Soura Mardpour, M.Sc.[2], Seyedeh Esmat Hosseini, M.Sc.[2, 11], Maryam Esmaeilzadeh, M.D., FACC., FASE.[12], Hakimeh Sadeghian, M.D.[3], Gholamreza Bahoush, M.D.[13], Ali Bassi, M.D.[14], Ahmad Amin, M.D.[15], Roghayeh Fazeli, M.D.[2], Yaser Sharafi, M.D.[10], Leila Arab, M.D.[2], Mansour Movahhed, M.D.[8], Saeid Davaran, M.D.[3], Narges Ramezanzadeh, B.Sc.[3], Azam Kouhkan, M.D.[2], Ali Hezavehei, M.D.[16], Mehrnaz Namiri, M.Sc.[2], Fahimeh Kashfi, M.Sc.[17], Ali Akhlaghi, M.Sc.[17], Fattah Sotoodehnejadnematalahi, Ph.D.[2], Ahmad Vosough Dizaji, M.D.[18], Hamid Gourabi, Ph.D.[19], Naeema Syedi, Ph.D.[20], Abdolhosein Shahverdi, Ph.D.[2], Hossein Baharvand, Ph.D.[2], Nasser Aghdami, M.D., Ph.D.[2*]

1.Department of Surgery, Baqiyatallah Hospital, Tehran, Iran
2. Department of Regenerative Medicine, Cell Science Research Center, Royan Institute for Stem Cell Biology and Technology, ACECR, Tehran, Iran
3. Research Department, Tehran Heart Center, Tehran University of Medical Sciences, Tehran, Iran
4. Department of Echocardiography, Baqiyatallah Hospital, Tehran, Iran
5. Department of Cardiology, Baqiyatallah Hospital, Tehran, Iran
6. Department of Cardiac Surgery, Lavasani Hospital, Social Security Organization, Tehran, Iran
7. Rajaie Cardiovascular Medical and Research Center, Iran University of Medical Sciences, Tehran, Iran
8. Department of Nuclear Medicine, Hasheminejad Hospital, Tehran University of Medical Sciences, Tehran, Iran
9. Transplantation Research Center, NRITLD, Masih Daneshvari Hospital, Shaheed Beheshti University of Medical Science, Darabad, Niavaran, Tehran, Iran
10. Department of Internal Medicine, Baqiyatallah Hospital, Tehran, Iran
11. Student Research Committee, School of Nursing and Midwifery, Shahid Beheshti University of Medical Sciences, Tehran, Iran
12. Echocardiography Research Center, Rajaie Cardiovascular Medical and Research Center, Iran University of Medical Sciences, Tehran, Iran
13. Department of Pediatrics, Ali Asghar Pediatric Hospital, Tehran University of Medical Sciences, Tehran, Iran
14. Department of Hematology and Oncology, Firoozgar Hospital, Iran University of Medical Sciences, Tehran, Iran
15. Department of Heart Failure and Transplantation, Fellowship in Heart Failure and Transplantation, Rajaie Cardiovascular Medical and Research Center, Iran University of Medical Sciences, Tehran, Iran
16. Department of Internal Medicine, Lavasani Hospital, Social Security Organization, Tehran, Iran
17. Department of Epidemiology and Reproductive Health, Reproductive Epidemiology Research Center, Royan Institute for Reproductive Biomedicine, ACECR, Tehran, Iran
18. Department of Reproductive Imaging, Reproductive Biomedicine Research Center, Royan Institute for Reproductive Biomedicine, ACECR, Tehran, Iran
19. Department of Genetics, Reproductive Biomedicine Research Center, Royan Institute for Reproductive Biomedicine, ACECR, Tehran, Iran
20. School of Pharmacy and Medical Sciences, Sansom Institute for Health Research, University of South Australia, South Australia, Australia

**Corresponding Address: P.O.Box: 16635-148, Department of Regenerative Medicine, Cell Science Research Center, Royan Institute for Stem Cell Biology and Technology, ACECR, Tehran, Iran*
Email: Nasser.aghdami@royaninstitute.org

#The first two authors equally contributed to this article.

Table S1: Comparison of the NYHA classification between groups at baseline and after 18 months of follow-up

Time/NYHA class	Placebo n=26	$CD133^+$ n=21	MNC n=30	P value
Baseline, n (%)				0.129
I	7 (26.9)	2 (9.5)	5 (16.7)	
II	16 (61.5)	12 (57.1)	13 (43.3)	
III	3 (11.5)	7 (33.3)	12 (40.0)	
IV	0.0	0.0	0.0	
18 months, n (%)				0.131
I	17 (70.8)	14 (70.0)	22 (91.7)	
II	7 (29.2)	6 (30.0)	2 (8.3)	
III	0.0	0.0	0.0	
IV	0.0	0.0	0.0	
Change, n (%)				0.178
Improvement≥1 class	13 (54.2)	17 (85.0)	18 (75.0)	
Unchanged	10 (41.7)	3 (15.0)	6 (25.0)	
Worsening≥1 class	1 (4.2)	0	0	

NYHA; New York Heart Association and MNC; Mononuclear Cells.

Table S2: Comparison of the end-point analyses of different variables between 6 and 18 months of follow-up by SPECT

Variables in groups	Baseline mean	6-month mean (SD)	18-month mean (SD)	Between-group differences (P value)		
				$CD133^+$ vs. placebo	MNC vs. placebo	$CD133^+$ vs. MNC
LVEF (%)	39.64			8.962 [0.011]*	6.917 [0.022]*	2.045 [0.562]
Placebo		39.69 (1.94)	38.37 (2.62)			
$CD133^+$		47.80 (3.00)	48.01 (3.29)			
MNC		47.03 (2.14)	42.10 (3.00)			
WMS	5.45			-1.268 [0.178]	-1.495 [0.083]	0.228 [0.814]
Placebo		4.19 (0.57)	5.14 (0.60)			
$CD133^+$		2.92 (0.75)	3.88 (0.74)			
MNC		2.69 (0.62)	3.65 (0.65)			
NV	2.90			-1.476 [0.001]*	-0.301 [0.443]	-1.175 [0.010]*
Placebo		2.12 (0.35)	1.89 (0.29)			
$CD133^+$		0.60 (0.45)	0.45 (0.34)			
MNC		1.96 (0.40)	1.55 (0.31)			
Dec. thickening	11.14			-3.748 [0.028]*	-3.552 [0.024]*	-0.196 [0.909]
Placebo		7.70 (1.14)	9.83 (1.16)			
$CD133^+$		4.85 (1.83)	5.19 (1.38)			
MNC		5.31 (1.26)	5.12 (1.31)			
PDS	14.21			-2.739 [0.206]	-1.751 [0.346]	-0.988 [0.643]
Placebo		8.18 (1.52)	10.20 (1.23)			
$CD133^+$		7.25 (2.33)	5.66 (1.53)			
MNC		8.42 (1.63)	6.47 (1.31)			

MNC; Mononuclear Cells, LVEF; Left ventricular ejection fraction, WMS; Wall motion score, NV; Non-viable segments, Dec. thickening; Decreased systolic wall thickening, PDS; Perfusion defect score, SPECT; Single Photon Emission Computed Tomography, and *; Significant difference between placebo and cell groups. Differences between group means are estimated by the Mixed model. The time by group interaction is not significant, so the estimated mean is related to both 6 and 18 month periods of time. Covariates in the model are evaluated for three groups at the baseline values that included: LVEF (39.64%), WMS (5.45), N.V (2.90), Dec. thickening (11.14), and PDS (14.21). The actual measurement of means in the three groups at baseline is listed in Table 3.

SPECT SCORING PAD

Grading		1: Mild Hypo<br>2: Moderate Hypo<br>3: Severe Hypo<br>4: Absent Perfusion	1: Viable<br>2: Non-V.	1:Hypokinesia<br>2:Akinesia<br>3:Dyskinesia	1:Mild Decrease<br>2:Mod Decrease<br>3:Severe Dec.
Segments		Perfusion Defect Score<br>Rest	Viability	Motion	Thickening
1	Basal	□1 □2 □3 □4	□1 □2	□1 □2 □3 □4	□1 □2 □3 □4
2	Basal Ant.Sep	□1 □2 □3 □4	□1 □2	□1 □2 □3 □4	□1 □2 □3 □4
3	Basal Inf.Sep	□1 □2 □3 □4	□1 □2	□1 □2 □3 □4	□1 □2 □3 □4
4	Basal Inferior	□1 □2 □3 □4	□1 □2	□1 □2 □3 □4	□1 □2 □3 □4
5	Basal Inf.Lat.	□1 □2 □3 □4	□1 □2	□1 □2 □3 □4	□1 □2 □3 □4
6	Basal Ant.Lat.	□1 □2 □3 □4	□1 □2	□1 □2 □3 □4	□1 □2 □3 □4
7	Mid. Anterior	□1 □2 □3 □4	□1 □2	□1 □2 □3 □4	□1 □2 □3 □4
8	Mid. Ant. Sep.	□1 □2 □3 □4	□1 □2	□1 □2 □3 □4	□1 □2 □3 □4
9	Mid. Inf. Sep.	□1 □2 □3 □4	□1 □2	□1 □2 □3 □4	□1 □2 □3 □4
10	Mid. Inferior	□1 □2 □3 □4	□1 □2	□1 □2 □3 □4	□1 □2 □3 □4
11	Mid. Inf. Lat.	□1 □2 □3 □4	□1 □2	□1 □2 □3 □4	□1 □2 □3 □4
12	Mid. Ant. Lat.	□1 □2 □3 □4	□1 □2	□1 □2 □3 □4	□1 □2 □3 □4
13	Apical	□1 □2 □3 □4	□1 □2	□1 □2 □3 □4	□1 □2 □3 □4
14	Apical Septal	□1 □2 □3 □4	□1 □2	□1 □2 □3 □4	□1 □2 □3 □4
15	Apical Inf.	□1 □2 □3 □4	□1 □2	□1 □2 □3 □4	□1 □2 □3 □4
16	Apical Lat.	□1 □2 □3 □4	□1 □2	□1 □2 □3 □4	□1 □2 □3 □4
17	Apex	□1 □2 □3 □4	□1 □2	□1 □2 □3 □4	□1 □2 □3 □4

Fig.S1: Scoring of different variables in 17 myocardial segments by SPECT. The analysis was performed by SPECT as previously described and according to 5 variables of perfusion defect score, viability, motion, systolic wall thickening and ejection fraction (not shown).
Ant; Anterior, Inf; Inferior, Lat; Lateral, Sep; Septal, and SPECT; Single Photon Emission Computed Tomography.
